# Quantitative evaluation of the central and local community-based home care policy in China based on the PMC Index model

**DOI:** 10.3389/fpubh.2025.1693612

**Published:** 2025-11-19

**Authors:** Shuhan Liang, Shiyu Rong

**Affiliations:** Management School, Minzu University of China, Beijing, China

**Keywords:** traditional Chinese’ population aging development, community-based home care policy, quantitative evaluation of policy, policy consistency, PMC-Index model

## Abstract

**Background:**

The community-based home care model is a vital strategy and pivotal response against global population aging. However, multidimensional empirical studies that quantitatively evaluate the design and consistency of community-based home care policies are scarce and have left a critical gap in understanding and optimizing CHC policies.

**Methods:**

This research introduces a novel evaluative framework combining text mining and the Policy Modeling Consistency (PMC) index model to quantitatively assess the consistency and design of 30 Chinese CHCPs issued by central and local government from 2021 to 2024.

**Results:**

The study yields two key original findings. First, it empirically identifies a core tension in multi-level governance: high longitudinal consistency coexists with significant horizontal disparities in local policy capabilities. Second, it pinpoints deficiencies in policy nature, content, and timeliness as primary constraints.

**Conclusion:**

The study’s primary theoretical contribution is to present the first empirical and quantitative analysis of CHCPs from a multidimensional level by using the PMC Index model. The primary practical contribution is that through a multidimensional consistency evaluation, the study offers specific, evidence-based optimized pathways for policymakers to enhance policy coherence as well as effectiveness and to bridge the gap between central planning and local implementation.

## 1 Introduction

The population aging and rising prevalence of chronic diseases driven by increasing life expectancy and declining fertility rates have emerged as critical demographic challenges, imposing unprecedented strains on healthcare systems worldwide (1–3). In response, nations are actively seeking to balance cost containment with the quality of care for older adults (4–6). This has catalyzed a paradigm shift towards community-based home care, a model strongly advocated by the World Health Organization to enable “aging in place” (7, 8). By allowing older individuals to receive care at their home and community with maximum independence (6), the model improves the quality of life of older adults, promotes social engagement, and alleviates the burdens on institutional care systems and public finances (6, 9–11). Consequently, the development and implementation of community-based home care policies (CHCPs) have become a national priority for many countries addressing the challenges of an aging population (12, 13).

China exemplifies this global trend, having rapidly transitioned into one of the world’s most severely aging societies. With 297 million people aged 60 or above, representing 21.1% of its population, the demand for effective older adult care solutions is urgent. The Chinese government has responded by issuing a series of CHCPs to promote the community-based home care model. Despite the substantial proliferation of research on CHCPs, the analysis and knowledge about the policy consistency level and characteristics of China’s CHCPs are insufficient. Existing studies have predominantly employed theoretical and conventional analytical approaches to examine policy characteristics (12), effectiveness (14) landscape (15, 16), practice (17) comparisons (18), and challenges (15). What is notably lacking is a rigorous, quantitative methodology capable of capturing the complexity of multi-level governance. Specifically, there is an absence of integrated analysis that simultaneously assesses the vertical consistency between central and local policies and the horizontal disparities in policy formulation across different local governments. This gap severely limits a nuanced understanding of policy efficacy and the dynamics of implementation (19, 20).

The Policy Modeling Consistency (PMC) Index model has emerged as a validated tool for quantitative policy evaluation (21). Its previous applications confirm its utility in assessing policy internal consistency (22–27). Moreover, in the older adult care and aging-related policy research, such as policies of digital access usage and literacy enhancement in the older adult population (28), China’s long-term care insurance policies (29–31), and policies for combining medical and nursing care (32), the PMC Index has also been widely used. Nevertheless, a significant shortcoming remains in its conventional application. Most studies employing the PMC Index model have confined their scope to a single-dimensional consistency analysis, failing to leverage its full potential for a stratified, multidimensional evaluation that can reveal the critical interplay between different administrative tiers. This methodological limitation has left the “national-local implementation gap” in policy research, particularly regarding aging policies, underexplored.

To address these interconnected gaps, the study is designed with three clear objectives. First, it develops a novel multidimensional evaluation framework based on the PMC Index model to quantitatively assess both the longitudinal and horizontal consistency of China’s CHCPs. Second, it empirically identifies the specific strengths and weaknesses inherent in these policies across different administrative levels. Finally, by introducing this dual-level comparison and stratified assessment, the study aims to enhance the methodological robustness of the PMC model itself and provide actionable, evidence-based guidance for policymakers to improve policy coherence and effectiveness in China’s aging response.

## 2 Method

### 2.1 PMC Index model

Based on the hypothesis of ‘Omnia Mobilis’, Estrada (21) proposed the Policy Consistency Index (PMC) model which is a multidisciplinary method. At present, the PMC has been widely used to analyze the internal consistency of policies and the development level of individual policies. Compared with other policy evaluation methods, the PMC Index model not only offers greater flexibility and scientific rigor in variable selection but also enables a multidimensional analysis of the internal strengths and weaknesses within policies.

In this study, the evaluation of community-based home care policies (CHCPs) was conducted through the following structured procedure:

(1) Construction of a multi-input–output table of the CHCPs.

Text mining techniques were applied to a selected corpus of policy documents to extract high-frequency keywords. Based on the structure of the Policy Modeling Consistency (PMC) Index model, the review of relevant literature, and the key words, the paper identified 9 primary variables and 54 secondary variables suitable for evaluating CHCPs, as shown in Table 1.

X~n

(2) Calculation of a multi-input–output table.

**Table 1:** The policy variables and evaluation criteria.

Primary variables	Sub-variables	Evaluation criteria	
Policy nature (X_1_)	Predict	X_1-1_	Whether the policy involves a description of the predictive nature, such as the institutional framework is basically determined, enhanced, and sound
	Supervise	X_1-2_	Whether the policy involves the supervision
	Proposal	X_1-3_	Whether the policy makes comments or recommendations
	Support	X_1-4_	Whether the policy provides effective support and measures
	Guide	X_1-5_	Whether the policy involves guiding content
	Describe	X_1-6_	Whether the policy involves detailed description
	Feedback	X_1-7_	Whether the policy has feedback channels for the corresponding problems
	Diagnose	X_1-8_	Whether the policy has a diagnosis of the existing problem
Policy types (X_2_)	Macro-policy	X_2-1_	Is the policy a macro policy
	Fundamental policy	X_2-2_	Is the policy a fundamental policy
	Specific policy	X_2-3_	Is the policy a specific policy
Policy audiences (X_3_)	Government	X_3-1_	Whether the target of policy action is the government and its relevant subordinate departments
	Community	X_3-2_	Whether the target of policy action is the community where older adults live
	Elders and family	X_3-3_	Whether the target of policy action are elders and their family
	Pension organization	X_3-4_	Whether the target of policy action are pension organizations
	Social organization	X_3-5_	Whether the target of policy action are the social organizations
Policy limitation (X_4_)	Long term	X_4-1_	Whether the policy prescription involves long-term planning (more than 5 years)
	Mid-term	X_4-2_	Whether the policy time limit involves medium-term planning (3–5 years)
	Short term	X_4-3_	Whether the policy time limit involve short-term planning (1–3 years)
Policy tool (X_5_)	Supply side	X_5-1_	Whether the policy plays a direct role in promoting it usually reflects the effective support of government funds, talents, facilities, technology, information and other aspects
	Environment	X_5-2_	Whether policies play an indirect role is usually reflected in the provision of a favorable policy environment through targets, plans, regulations, finance and taxation, including target planning, financial services, tax incentives, regulations, and strategic measures.
	Demand side	X_5-3_	Whether the policy plays a pulling role to release the demand for policy objectives and reduce external interference, including service outsourcing
Policy trend (X_6_)	Improve construction of system	X_6-1_	Whether the policy is inclined to improve the construction of community home care standard system
	Improve basic security	X_6-2_	Whether the policy is inclined to improve the basic security of community home care
	Improve service function	X_6-3_	Whether the policy is inclined to help the community home care function
	Clear responsibility	X_6-4_	Whether the policy is inclined to clarify the main responsibility of community home care
	Strengthen management	X_6-5_	Whether the policy is inclined to strengthen the management of community home care service
	Improve service efficiency	X_6-6_	Whether the policy is inclined to enhance the efficiency of community home care service
	Improvement environment	X_6-7_	Whether the policy is inclined to strengthen the improvement of community home care environment
	Encourage	X_6-8_	Whether the policy is inclined to encourage the improvement of community home care services
	Enhance standard	X_6-9_	Whether the policy is inclined to lead the community home care service norms
	Collaborative management	X_6-10_	Whether the policy is inclined to strengthen cooperation and information sharing among offices
	Innovation	X_6-11_	Whether the policy is inclined to strengthen the innovation of community home care service and digital care
Policy content (X_7_)	Medical care	X_7-1_	Whether the policy involves the medical care of older adults at home in the community
	Home care	X_7-2_	Whether the policy involves the care responsibility, care service and care function of older adults at home in the community
	Talent	X_7-3_	Whether the policy involves the construction of talent team for community home care
	Financial support	X_7-4_	Whether the policy involves financial support and economic help for community home care
	Mental comfort	X_7-5_	Whether the policy involves the spirit and entertainment of older adults at home in the community
	Supervision	X_7-6_	Whether the policy involves the law and supervision and protection of community home care
	Propaganda	X_7-7_	Whether the policy involves the correct publicity and guidance of community home care
	Promotion	X_7-8_	Whether the policy involves the upgrading of community home care facilities
	Pension industry	X_7-9_	Whether the policy involves the development of community home care industry
	Pension production	X_7-10_	Whether the policy involves the development of community home care products
	Digital intelligence	X_7-11_	Whether the policy involves digital intelligence of community home care
Policy evaluation (X_8_)	Clear objectives	X_8-1_	Whether the policy has clear and quantifiable indicators
	Sufficient basis	X_8-2_	Whether the policy is formulated in accordance with relevant laws and regulations and central documents
	Well-planned	X_8-3_	Whether the policy content is perfect and detailed
	Scheme of science	X_8-4_	Whether the policy plan is scientific and reasonable
	Accord with true situation	X_8-5_	Whether the policy is in line with the national conditions
Policy area (X_9_)	Economy	X_9-1_	Whether the policy involves the economic and financial area
	Social	X_9-2_	Whether the policy involves the area of society and social institutions
	Science and technology	X_9-3_	Whether the policy involves the science and technology area
	Talent	X_9-4_	Whether the policy involves the talent area
	Service	X_9-5_	Whether the policy relates to the service area

According to the content of each policy text, secondary variables were denoted by “1” or “0” and assigned a binary score: if the sub-variable could fit into the evaluation criteria, it was assigned a value of 1; otherwise, it was denoted 0.

X={XR:[0,1]},

(3) Calculation of primary variables.

The value of each primary variable was derived by aggregating the scores of its corresponding secondary variables, using the formula:

$$X_{t}=[\sum_{j=1}^{n}\frac{X_{\mathrm{tj}}}{\left(X_{\mathrm{tj}}\right)}],t=1,2,3\ldots \infty$$

(4) Calculation of the PMC Index.

The overall PMC Index for each policy was computed by summing the scores of the nine primary variables, providing a quantitative measure of policy consistency.

$$\mathrm{PMC}=[\begin{array}{l} X_{1}(\sum_{n=1}^{8}\frac{x_{1n}}{5})+X_{2}(\sum_{t=1}^{3}\frac{x_{2t}}{3})+X_{3}(\sum_{a=1}^{5}\frac{x_{3a}}{5})\\ +X_{4}(\sum_{I=1}^{3}\frac{x_{4I}}{3})+X_{5}(\sum_{t=1}^{3}\frac{x_{5t}}{3})+X_{6}(\sum_{t=1}^{11}\frac{x_{6t}}{11})\\ +X_{7}(\sum_{c=1}^{11}\frac{x_{7c}}{11})+X_{8}(\sum_{e=1}^{5}\frac{x_{8e}}{5})+X_{9}(\sum_{a=1}^{5}\frac{x_{9a}}{5})+X_{10} \end{array}]$$

(5) Construction of the PMC surface diagram.

To visually represent the strengths and weaknesses of each CHCPs, the values of the nine primary variables were arranged into a 3 × 3 matrix, which was then used to generate a PMC surface diagram. This facilitates an intuitive comparison of policy profiles and highlights areas requiring improvement.

$$\mathrm{PMC}\;\text{surface}=[\begin{array}{ccc} X_{1} & X_{2} & X_{3}\\ X_{4} & X_{5} & X_{6}\\ X_{7} & X_{8} & X_{9} \end{array}]$$

Referring to the PMC Index rating criteria proposed by Ruiz Estrada (21) and combining it with the actual situation of CHCPs, the PMC Index rating criteria of CHCPs are classified into the following four levels: Perfect consistency; Good consistency; acceptable consistency; low consistency in Table 2.

**Table 2:** Classification of the PMC index of CHCPs.

PMC Index	9 ~ 7	6.99 ~ 5	4.99 ~ 3	2.99 ~ 0
Evaluation	Perfect consistency (PC)	Good consistency (GC)	Acceptable consistency (AC)	Low consistency (LC)

### 2.2 Sample selection

This study aims to examine and evaluate community-based home care policies (CHCPs) promulgated by the Chinese government. To assemble a robust corpus of policy texts, four retrieval strategies were systematically implemented.

1. to enhance suitability, the China National Knowledge Infrastructure (CNKI) policy database was queried using key terms such as “community-based home care” and “older adults home care.” Redundant documents were excluded to preserve sample uniqueness and analytical validity.
2. to ensure comprehensiveness and facilitate multi-dimensional analysis, policy documents were selected across multiple tiers of governance, encompassing 10 central-level and 20 local-level policies. The provincial policies were drawn from the 10 regions with the highest aging severity indices to capture critical geographic variation, as shown in Table 3.
3. to ascertain temporal relevance and authenticity, all selected policies were issued between 2021 and 2024 and retrieved from authoritative sources, including the PKULAW database, CNKI, and official government portals.
4. to secure diversity and representativeness, first, the diversity of the source of the policy was conserved. The sample incorporates variation across issuing entities—including policies promulgated by individual government agencies, multi-department collaborations, and different administrative levels. In selecting local policies, attention was paid to achieving geographical balance, covering eastern, western, southern, northern, and central regions, while also incorporating variations in economic development levels and differences in policy intensity. Second, the diversity of policy types was considered. These policy texts cover operational guidelines, notices, announcements, temporary regulations, and programs, as shown in Table 4.

**Table 3:** The ranking of province of aging index in China.

Rate	Area name	Aging index Over65(%)
1	Liaoning Province	21.06
2	Shanghai	19.56
3	Chongqing	18.91
4	Heilongjiang Province	18.81
5	Jilin Province	18.65
6	Jiangsu Province	18.46
7	Sichuan Province	18.46
8	Tianjin	17.91
9	Shandong Province	17.43
10	Hubei Province	16.95

**Table 4:** China’s central and local CHCPs samples for PMC Index model (partial).

Level	Code	Policy code	Name	Time	Issuing authority
Central	P1		Outline of the 14th Five-Year Plan for the National Economic and Social Development of the People’s Republic of China and the Long-Range Objectives through the Year 2035	2021.03.11	NPC
	P2	The State Council issued in 2021 No. 35	Notice of the State Council on Printing and Distributing the ‘14th Five-Year Plan’ for the Development of National Aging Undertakings and the Pension Service System	2021.12.3	The State Council
	…	…	…	…	…
	P10	National Development and Reform Commission issued in 2022 No.1356	The National Development and Reform Commission and other departments issued a notice on the ‘Several Policies and Measures for Relief and Support of the Older Care Service Industry’	2022.08.29	National Development and Reform Commission
Local	Q1	Liaoning Civil Affairs issued in 2021 No. 28	Several measures to establish and improve the comprehensive supervision system for pension services and promote the high-quality development of pension services	2021.08.11	Liaoning Provincial Department of Civil Affairs
	Q2	General Office of Liaoning Provincial People’s Government issued in 2023 No. 11	Notice of the General Office of the People’s Government of Liaoning Province on Printing and Distributing the Implementation Plan for Promoting the Construction of the Basic Pension Service System in Liaoning Province	2023.09.17	General Office of Liaoning Provincial People’s Government
	…	…	…	…	…
	Q20	Hubei Civil Affairs issued in 2022 No. 35	Notice of the Hubei Provincial Department of Civil Affairs, the Hubei Provincial Department of Natural Resources, and the Hubei Provincial Department of Housing and Urban–Rural Development on Printing and Distributing the Implementation Measures for the “Four Synchronizations” of Older Service Facilities in New Residential Quarters in Hubei Province (for Trial Implementation)	2022	Hubei Provincial Department of Civil Affairs

### 2.3 Word frequency statistics

**Figure 1:**
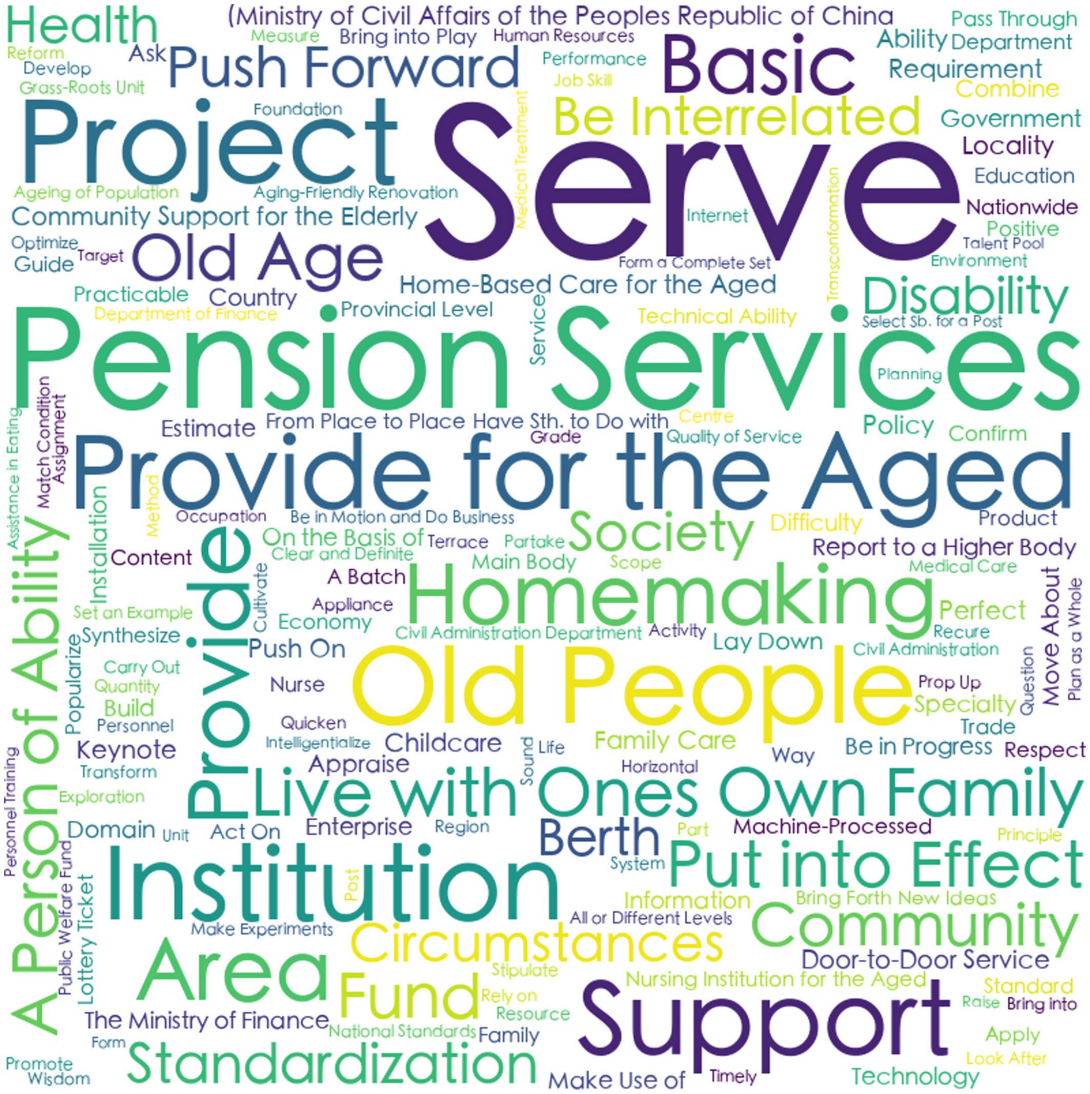
The cloud map of central f high-frequency words.

**Figure 2:**
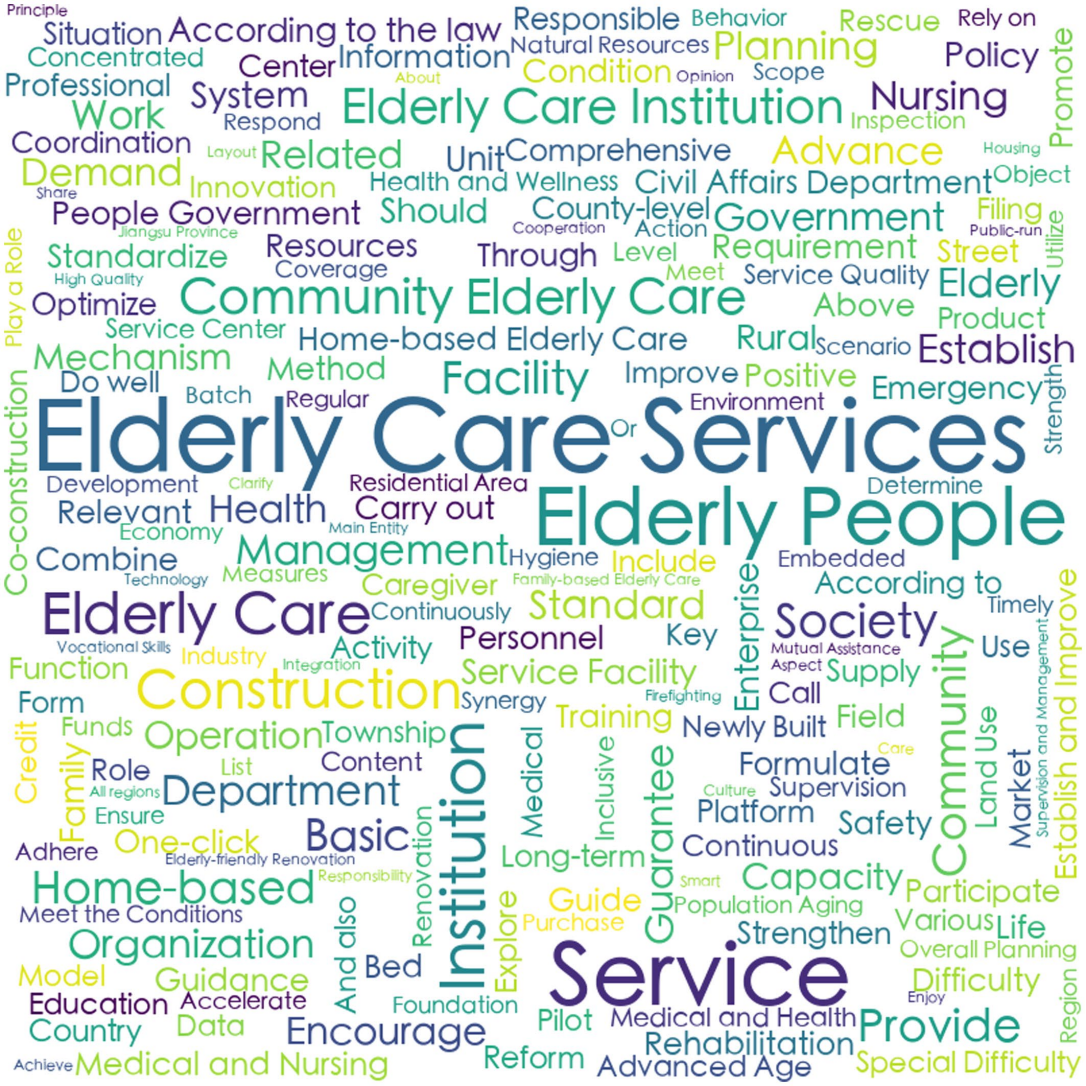
The cloud map of local f high-frequency words.

**Figure 3:**
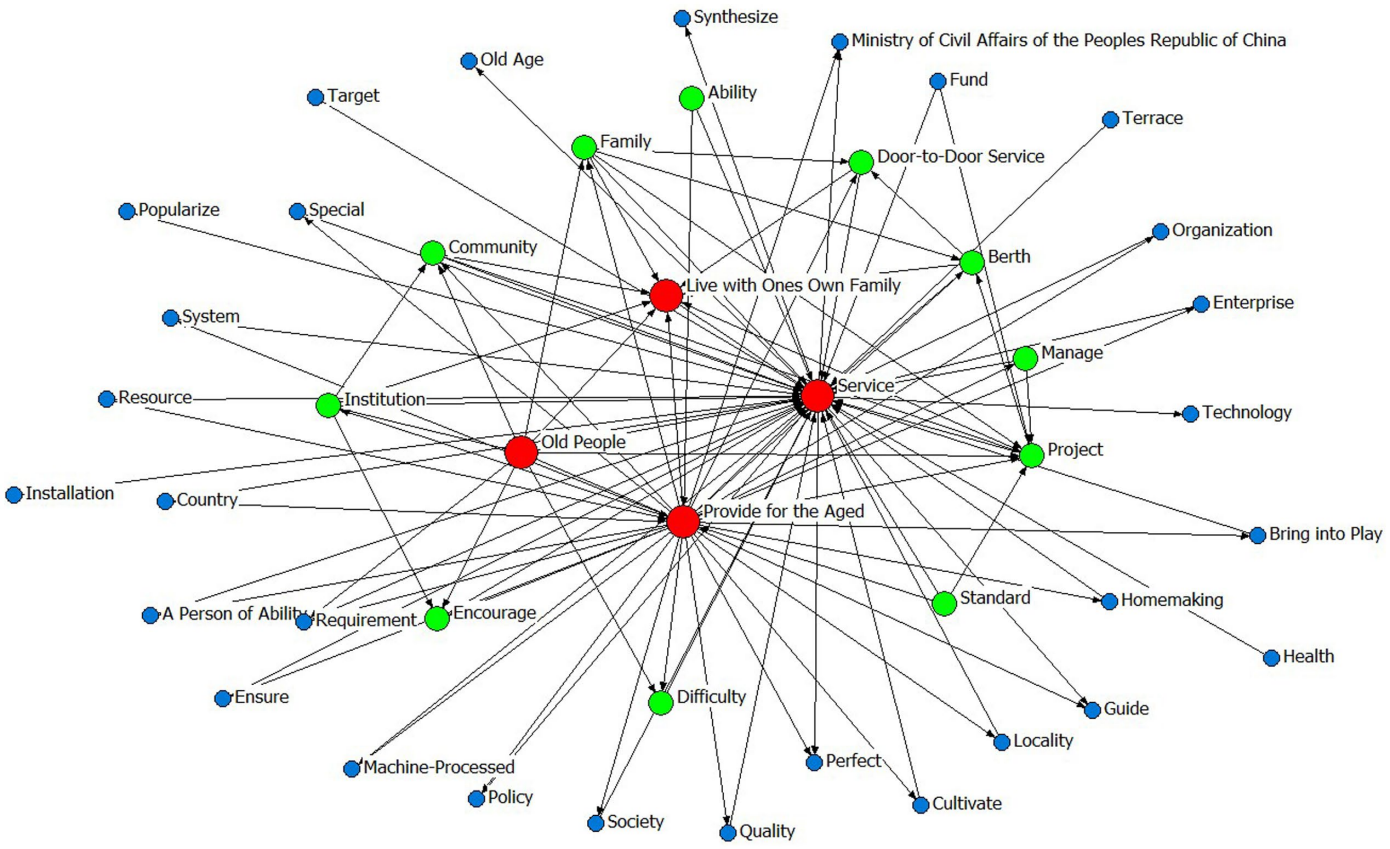
The social network map of central high-frequency words.

**Figure 4:**
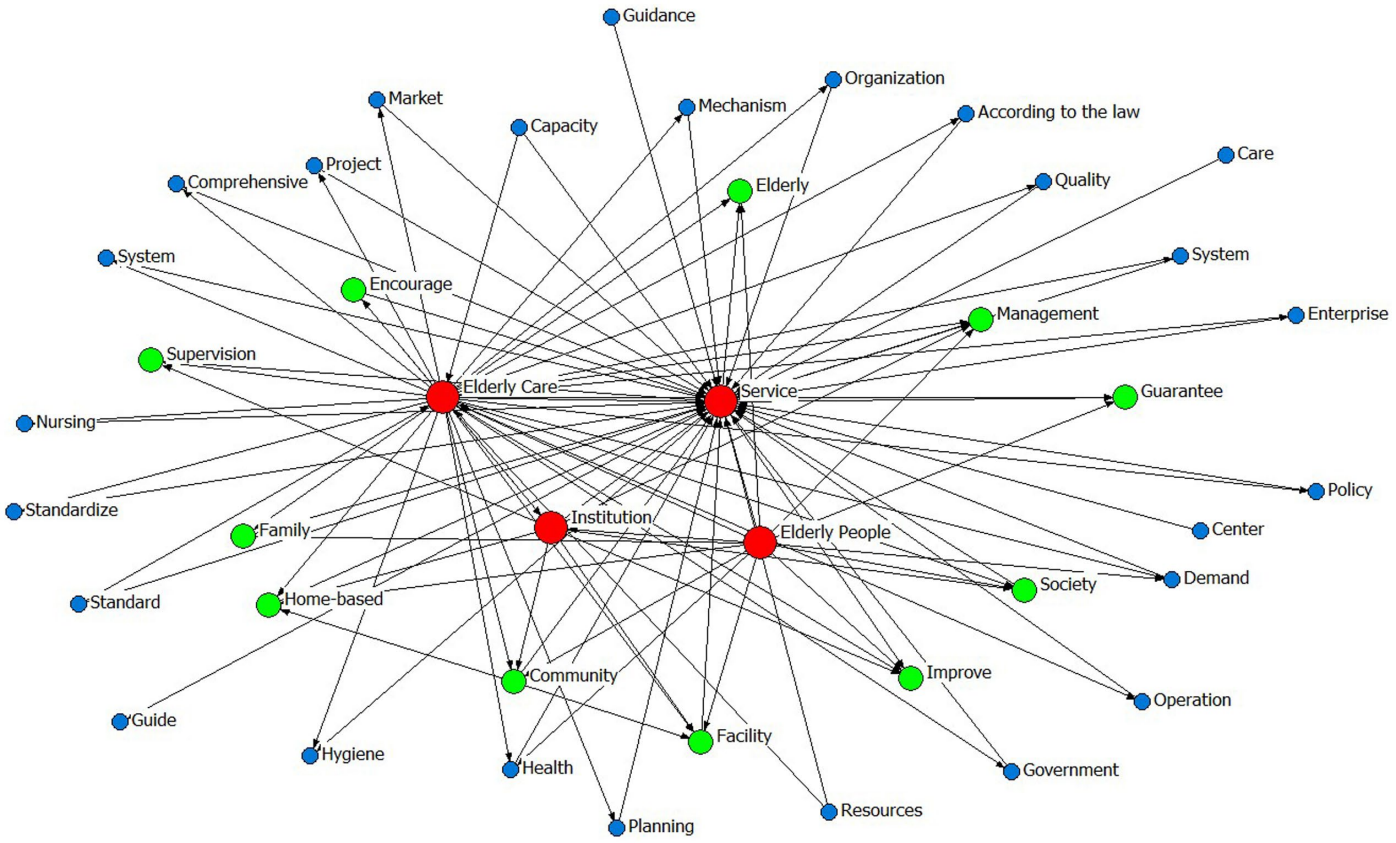
The social network map of local high-frequency words.

Prior to variable determination, policy text mining was systematically conducted through the following analytical sequence:

1. Convert the collected texts of 10 central and 20 local CHCPs into a format that can be recognized by the processing software.
2. Import the policy texts into the software ROSTCM 6.0 for automated word segmentation and lexical extraction.
3. Count the frequency of word segmentation results of central and local CHCPs.
4. Refine the analytical output by eliminating stop words, along with high-frequency nouns, quantifiers, and verbs that lack discriminative textual features or substantive relevance to the research focus.
5. The remaining meaningful terms were then ranked by frequency, with the top 50 representative keywords from both central and local policies retained for subsequent analysis (Table 5).
6. A co-occurrence matrix was constructed to identify thematic priorities and conceptual linkages within the policy texts, thereby elucidating the semantic relationships between keywords and their contextual frameworks. Co-occurrence matrices derived from these keywords were visualized as semantic network diagrams (Figures 1–4), revealing thematic structures and conceptual relationships within the policy corpus.

**Table 5:** The top 50 most frequent keywords of the central and local CHCPs.

Central policies			Local policies		
Serial number	Key words	Frequency	Serial number	Key words	Frequency
1	Serve	417	1	Older Care Services	1,529
2	Older care services	354	2	Service	1,114
3	project	231	3	Older People	892
4	Older people	198	4	Institution	506
5	Institution	187	5	Development	492
6	Older care	182	6	Older Care	441
7	Support	175	7	Construction	383
8	Standard	172	8	Community Older Care	327
9	Construction	149	9	Institution	310
10	Development	139	10	Society	300
11	Provide	119	11	Support	299
12	Area	119	12	Strengthen	295
13	Homemaking	117	13	Community	291
14	Basic	115	14	Provide	289
15	Strengthen	107	15	Home-Based Care for the Aged	282
16	Home-based	102	16	Facility	274
17	Put into effect	93	17	Supervision	267
18	Older adults	93	18	Basic	261
19	Carry out	89	19	Carry out	236
20	A person of ability	87	20	Department	223
21	Work	87	21	Management	222
22	Fund	86	22	Older adults	209
23	Management	85	23	Organization	203
24	Community	80	24	Establish	203
25	Encourage	79	25	Standardization	202
26	Promote	74	26	Improvement	201
27	Enhance	72	27	Work	200
28	Standardization	70	28	Nursing	199
29	Society	68	29	Planning	196
30	Organization	65	30	Promote	184
31	Circumstances	63	31	Encourage	184
32	Be Interrelated	63	32	Related	181
33	Berth	61	33	Guarantee	178
34	Disability	60	34	Enhance	178
35	Health	58	35	Health	175
36	Family	56	36	Advance	172
37	Home-based care for the aged	56	37	System	166
38	Community support for the older adults	53	38	Requirement	166
39	Improvement	52	39	Government	165
40	Cultivate	52	40	Implement	163
41	System	51	41	Operation	157
42	Locality	51	42	Care	152
43	Target	51	43	Mechanism	149
44	Advance	50	44	System	148
45	Door-to-door service	49	45	Ability	145
46	Ability	48	46	Comprehensive	142
47	Keynote	48	47	Unit	137
48	Policy	48	48	Service Facility	137
49	Requirement	46	49	Policy	134
50	Childcare	46	50	Family	134

In the social network maps, keywords are represented as circular nodes, with connecting lines indicating their co-occurrence relationships. Central policy keywords such as “Provide for the aged,” “Service,” and “Old people” occupy core positions, reflecting their high frequency and broad semantic connectivity. Related concepts like “Door-to-door Service,” “Community,” and “Standard” form a cohesive cluster, indicating strong associative relevance. Similarly, local policies highlight “Older care service,” “older adults people,” and “older adults care” as central lexical nodes, with “Family,” “Home-based,” “Community,” and “Facility” comprising a closely linked subgroup, as shown in Table 5 and Figures 1–4.

### 2.4 Definition of variable and identification of parameter

To define the variables and build the evaluation index system for China’s CHCPs, the specific ideas are as follows.

1. The nine9 primary variables were designed by referring to existing studies (20–32), including (X1) policy nature; (X2) policy types; (X3) policy audiences; (X4) policy limitation; (X5) policy tool; (X6) policy trend; (X7) policy content; (X8) policy evaluation; and (X9) policy area.
2. The secondary variables were designed based on existing literatures and frequency-based keyword analysis. Building upon the frequency-based keyword analysis and semantic network mapping, the paper systematically identified lexical patterns reflective of core CHCPs priorities. These empirically grounded terms subsequently informed the operationalization of secondary variables within the PMC Index framework. For instance, frequently co-occurring terms including “older adults,” “society,” “organization,” and “family,” collectively shaped the sub-variable structure for X3 (policy audience). Similarly, recurring lexemes such as “construction” and “basic” guided the formulation of sub-variables X6-1 (system construction improvement) and X6-2 (basic security enhancement), while the consistent presence of “home care” across policy levels substantiated its inclusion as sub-variable X7-2.
3. The determination of the value of each secondary indicator. The value of the second-level indicator is assigned 0 or 1. Taking X4 as an example, the policy limitation X4 consists of three second-level indicators (X4-1 long term, X4-2 mid-term, and X4-3 short term) to evaluate which type of time limit this policy falls into. If the policy belongs to X4-1 and X4-1 is equal to 1; otherwise, its value is 0, which shows X4-1 = 1, X4-2 = X4-3 = 0. Moreover, if the policy involves all goals, it shows X4-1 = X4-2 = X4-3 = 1. The indicators’ definitions and parameter settings of the central and local CHCPs follow the same rules, as shown in Table 1 (21, 22, 27, 33–37).

### 2.5 Establishment of multi-input–output matrix and measurement of PMC Index

After the parameters of variables were determined, the multi-input–output matrices of the 10 central CHCPs and 20 local CHCPs were constructed, as shown in Table 6. The PMC Index value of each policy was calculated. In addition, the consistency score for the central CHCPs and local CHCPs is shown in Tables 7, 8.

**Table 6:** The multi-input–output matrix of the central and local policies (partial).

Classification	Primary variables	Sub-variables	Policies									
Central policy			P_1_	P_2_	P_3_	P_4_	P_5_	P_6_	P_7_	P_8_	P_9_	P_10_
	X_1_	X_1-1_	1	1	1	1	0	0	1	0	1	0
		X_1-2_	1	0	1	1	0	0	1	0	1	0
		X_1-3_	1	1	1	1	1	1	1	1	1	1
		X_1-4_	1	1	1	1	1	1	1	1	1	1
		X_1-5_	1	1	1	1	1	1	1	1	1	1
		X_1-6_	0	0	0	1	0	0	1	1	0	0
		X_1-7_	0	0	0	1	1	1	1	1	1	1
		X_1-8_	1	1	0	1	0	0	0	0	0	0
	X_2_	X_2-1_	1	0	0	1	0	0	0	0	0	0
		X_2-2_	0	1	1	0	0	0	0	0	1	0
		X_2-3_	0	0	0	0	1	1	1	1	0	1
	…	…	…	…	…	…	…	…	…	…	…	…
	X_8_	X_8-1_	1	1	1	1	1	1	1	1	1	1
		X_8-2_	1	1	1	1	1	1	1	1	1	1
		X_8-3_	1	1	1	1	1	1	1	1	1	1
		X_8-4_	1	1	1	1	1	1	1	1	1	1
		X_8-5_	1	1	1	1	1	1	1	1	1	1
	X_9_	X_9-1_	1	1	1	1	1	1	1	1	0	1
		X_9-2_	1	1	1	1	1	1	1	1	1	1
		X_9-3_	1	1	1	1	1	1	1	1	1	1
		X_9-4_	1	1	1	1	1	1	1	1	1	0
		X_9-5_	1	1	1	1	1	1	1	0	1	1
Local policy			Q_1_	Q_2_	Q_3_	Q_4_	…	Q_16_	Q_17_	Q_18_	Q_19_	Q_20_
	X_1_	X_1-1_	1	1	1	1	…	1	1	1	1	0
		X_1-2_	1	0	0	1	…	1	1	1	1	1
		X_1-3_	0	1	1	1	…	0	1	0	1	0
		X_1-4_	1	1	1	1	…	1	1	1	1	1
		X_1-5_	0	1	1	1	…	1	1	1	1	1
		X_1-6_	1	1	1	0	…	1	0	1	1	1
		X_1-7_	1	1	0	1	…	0	1	1	1	1
		X_1-8_	1	1	1	1	…	0	0	0	1	0
	X_2_	X_2-1_	0	0	0	1	…	0	1	0	1	0
		X_2-2_	0	1	0	0	…	0	0	0	0	0
		X_2-3_	1	0	1	0	…	1	0	1	0	1
	…	…	…	…	…	…	…	…	…	…	…	…
	X_8_	X_8-1_	1	1	1	1	…	1	1	1	1	1
		X_8-2_	1	1	1	1	…	1	1	1	1	1
		X_8-3_	1	1	1	1	…	1	1	1	1	1
		X_8-4_	1	1	1	1	…	1	1	1	1	1
		X_8-5_	1	1	1	1	…	1	1	1	1	1
	X_9_	X_9-1_	1	1	0	1	…	1	1	0	1	0
		X_9-2_	1	1	1	1	…	1	1	1	1	1
		X_9-3_	0	1	1	1	…	1	1	0	1	0
		X_9-4_	0	1	0	1	…	1	1	0	1	0
		X_9-5_	1	1	1	1	…	1	1	1	1	1

**Table 7:** The PMC Index value and rating of the 10 central CHCPs.

Index	P_1_	P_2_	P_3_	P_4_	P_5_	P_6_	P_7_	P_8_	P_9_	P_10_	Mean
X_1_	0.75	0.63	0.63	1	0.5	0.5	0.88	0.63	0.75	0.5	0.68
X_2_	0.33	0.33	0.33	0.33	0.33	0.33	0.33	0.33	0.33	0.33	0.33
X_3_	1	1	1	0.8	1	1	0.8	1	1	1	0.96
X_4_	0.33	0.33	0.33	0.33	0.33	0.33	0.33	0.33	0.33	0.33	0.33
X_5_	1	1	1	0.67	0.67	0.67	0.67	0.67	1	0.33	0.77
X_6_	1	1	0.91	1	0.82	0.82	0.73	0.91	1	0.73	0.89
X_7_	1	1	1	1	0.73	0.91	0.73	0.91	0.82	0.55	0.86
X_8_	1	1	1	1	1	1	1	1	1	1	1
X_9_	1	1	1	1	1	1	1	0.8	0.8	0.8	0.94
Total PMC Index	7.42	7.29	7.2	7.13	6.38	6.56	6.46	6.58	7.03	5.57	6.76
Policy-level	PC	PC	PC	PC	GC	GC	GC	GC	PC	GC	GC
Rating	1	2	3	4	9	7	8	6	5	10	/

**Table 8:** The PMC Index value and rating of the 20 local CHCPs.

Index	Q_1_	Q_2_	Q_3_	Q_4_	Q_5_	Q_6_	Q_7_	Q_8_	Q_9_	Q_10_	Q_11_	Q_12_	Q_13_	Q_14_	Q_15_	Q_16_	Q_17_	Q_18_	Q_19_	Q_20_	Mean
X_1_	0.75	0.88	0.75	0.88	0.88	0.75	0.88	0.63	0.63	0.63	0.75	0.63	0.75	0.88	0.63	0.63	0.75	0.75	1.00	0.63	0.75
X_2_	0.33	0.33	0.33	0.33	0.33	0.33	0.33	0.33	0.33	0.33	0.33	0.33	0.33	0.33	0.33	0.33	0.33	0.33	0.33	0.33	0.33
X_3_	0.8	1	1	1	1	1	1	0.8	1	0.8	1	0.8	1	1	0.6	1	1	1	1	1	0.94
X_4_	0.33	0.33	0.33	0.33	0.33	0.33	0.33	0.33	0.33	0.33	0.33	0.33	0.33	0.33	0.33	0.33	0.33	0.33	0.33	0.33	0.33
X_5_	0.67	0.67	1.00	0.67	0.67	1.00	0.67	0.67	1.00	0.67	0.67	0.67	0.67	0.33	0.33	0.33	1.00	0.67	0.67	0.67	0.68
X_6_	0.64	0.91	0.64	1.00	1.00	0.91	1.00	0.64	0.73	1.00	0.64	0.73	0.73	0.45	0.27	0.27	0.91	0.36	1.00	0.36	0.71
X_7_	0.18	0.73	0.45	1.00	0.82	0.55	0.73	0.64	0.73	0.73	0.64	0.36	0.36	0.36	0.64	0.45	0.82	0.27	0.82	0.18	0.57
X_8_	1	1	1	1	1	1	1	1	1	1	1	1	1	1	0.8	1	1	1	1	1	0.99
X_9_	0.6	1	0.6	1	1	1	1	0.4	1	1	1	0.6	1	0.8	0.4	1	1	0.4	1	0.4	0.81
Total PMC Index	5.30	6.84	6.11	7.21	7.03	6.87	6.94	5.43	6.75	6.49	6.36	5.45	6.17	5.49	4.33	5.35	7.14	5.12	7.15	4.90	6.12
Policy-level	GC	GC	GC	PC	PC	GC	GC	GC	GC	GC	GC	GC	GC	GC	AC	GC	PC	GC	PC	AC	GC
Rating	17	7	12	1	4	6	5	15	8	9	10	14	11	13	20	16	3	18	2	19	/

## 3 Results

### 3.1 Longitudinal consistency evaluation of central-local CHCPs

The analysis of high-frequency words reveals both convergence and divergence in thematic focus between central and local community-based home care policies (CHCPs). As shown in Table 5, 68% of high-frequency words overlap, indicating substantial policy alignment, while the remaining 32% reflect level-specific emphases that local policies derive from their own status quo, strengths, and problems.

First, shared high-frequency terms cluster into four thematic categories: (1) Target Beneficiaries or Audiences: Terms such as “Older People,” “Community,” “Institution,” and “Family” highlight the multi-stakeholder nature of CHCPs implementation, underscoring the need for collaborative engagement across sectors. (2) Policy Objectives: Keywords like “Standardization,” “Home-based,” and “Health” illustrate a common aim to establish a systematic, foundational, and health-oriented aged care system. (3) Service Content: Phrases including “Older Care Services,” “Home-Based Care,” and “Community Support” reflect a unified focus on delivering home and community-based services to meet the diverse needs of older adults. (4) Implementation Pathways: Verbs such as “Strengthen,” “Promote,” and “Encourage” signal shared strategies aimed at amplifying policy execution and service quality.

Secondly, beyond these commonalities, distinctive keywords reveal differentiated roles. Central policies emphasize terms like “Project,” “Fund,” and “Cultivate,” reflecting a macro-level focus on program design, financial allocation, and talent development. In contrast, local policies prioritize terms such as “Implementation,” “Mechanism,” and “Service Facility,” highlighting their role in operationalizing services and adapting frameworks to local conditions.

Finally, the semantic networks of central and local policies provide longitudinal consistency, with direct examples shown in Figures 3, 4. The semantic analysis reveals that central policies are structured around core concepts including “service,” “old people,” “provided for the aged,” and “live with own family,” indicating a coherent focus on enabling aging in place through service provision for older adults. A strongly aligned lexical pattern is observed in local policies, where high-frequency terms such as “older adults people,” “service,” and “institution” reflect a high degree of conceptual consistency with central directives. This convergence in core terminology underscores a unified policy orientation across administrative levels regarding target beneficiaries and primary service objectives. Further analysis of sub-core keywords shows that among 11 recurring thematic nodes, seven share semantic equivalence, representing 60% conceptual overlap. This substantial alignment coexists with localized variations in implementation-related terminology, illustrating contextual adaptations in operational strategies while maintaining strategic coherence.

In summary, the keyword patterns demonstrate a distinct top-down alignment within China’s intergovernmental system, where semantic consistency in core dimensions reflects hierarchical integration, while peripheral lexical variations reveal adaptive implementation. These findings not only elucidate the structural drivers of community-based aging initiatives but also offer an empirically grounded basis for defining evaluative dimensions within policy consistency frameworks such as the PMC Index.

### 3.2 Horizontal consistency evaluation of the central CHCPs

The result of the PMC Index shows that the average PMC Index of central CHCPs is 6.76, illustrating that the central CHCPs are equipped with “Good” consistency, as shown in Table 7. In contrast, the average PMC Index of 20 local CHCPs is 6.12, illustrating that the local CHCPs are also equipped with “Good,” as shown in Table 8, but this is inferior to the central policies.

From the horizontal perspective of the same level of policies, among the central policies, half of the policies (P1, P2, P3, P4, P9) demonstrate “Perfect” consistency (scores 7–9), while the remaining half (P5–P8, P10) maintain “Good” levels (scores 5–6.99). High performance is driven by policy audience (X3, 0.96), policy trend (X6, 0.89), policy content (X7, 0.86), policy evaluation (X8, 1.00), and policy area (X9, 0.94). However, weaker scores in policy nature (X1, 0.68), policy type (X2, 0.33), and policy timeliness (X4, 0.33) constrain overall effectiveness.

The scores in key variables X3, X6, X7, X8, and X9 reflect well-articulated policy audiences, comprehensive sectoral trends, clearly defined strategic content, and well-designed evaluation and areas within central-level policy texts. For example, P2 establishes an institutional framework to address population aging through a coordinated home-community-institution service network and integrated healthcare-wellness systems. The policy emphasizes multi-stakeholder collaboration to develop accessible, life-oriented, and health-focused older adult services, incorporating specific measures such as facility optimization, diversified professional support, smart eldercare technologies, and age-friendly social initiatives. This comprehensive approach demonstrates strength in X3, X6, X7, X8, and X9.

However, the weaker score of central policies was contributed by X1, X2, and X4. The policy nature (X1) achieves an overall low score of 0.68, reflecting significant deficiencies in descriptive, diagnostic, supervision, prediction, and feedback components across central-level policies. Specifically, 70% of policies lack descriptive and diagnostic elements, while 50% omit supervision, which undermines formulation precision and implementation effectiveness. Forty percent neglect prediction, and 30% lack feedback. These omissions in core policy attributes compromise both the communicative clarity and operational efficacy of otherwise macro-strategic directives.

Similarly, policy type (X2) achieves an equally low score (0.33), indicating a predominance of qualitative macro-objectives such as improving and perfecting the basic older adult service system and building an older adult service system over specific operational plans. While these broad goals articulate strategic vision, the absence of concrete implementation mechanisms often reduces them to declarative statements with limited practical impact. The policy limitation (X4) scores notably low (0.33), reflecting a structural overreliance on medium-term frameworks with insufficient short- and long-term planning. This temporal imbalance constrains foresight, continuity, and diversity in policy design and implementation.

### 3.3 Horizontal consistency evaluation of the local CHCPs

At the local level, the average PMC Index of 20 local CHCPs is 6.12, illustrating that the local CHCPs are equipped with “Good,” as shown in Table 8. Among them, 4 policies (Q4, Q5, Q17, and Q19) are of “Perfect” consistency, accounting for 20%. Fourteen policies (Q7, Q6, Q2, Q9, Q10, Q11, Q13, Q3, Q14, Q12, Q8, Q16, Q1, and Q18) are of “Good consistency,” accounting for 70% of the total. Two policies (Q20 and Q15) are “Acceptable,” accounting for 10%.

Strong performance is observed in policy audience (X3, 0.94), Policy evaluation (X8, 0.99), and policy area (X9, 0.81). Taking Q19 as an example, this document actively responds to the national strategy on population aging by establishing mechanisms to improve the basic older adult care system and accelerate the development of an integrated home–community–institution service network coordinated with healthcare and wellness provisions. The policy systematically outlines the status of older adult care services in Hubei Province, diagnoses existing challenges, sets objectives aligned with national strategic priorities, and delineates concrete implementation measures. With its comprehensive content, robust evaluative framework, and multi-sectoral coverage, this policy represents a well-structured and systematically designed regulatory instrument.

Whereas policy content (X7, 0.57), policy nature (X1, 0.75), and policy limitation (X4) represent key limitations. The (X1) achieves an overall moderate score of 0.75 but conceals significant internal variations. Among the 20 local-level policies analyzed, seven documents (Q8, Q9, Q10, Q12, Q15, Q16, Q20) scored notably lower at 0.63, primarily due to deficiencies in descriptive, feedback, and diagnostic functions. This omission, specifically the lack of detailed contextual description, structured feedback mechanisms, and systematic problem diagnosis, undermines both the evidential basis during policy formulation and the adaptive capacity during implementation, ultimately compromising policy legitimacy and operational responsiveness.

The policy content (X7) records a relatively low average score of 0.57, indicating limited comprehensiveness and thematic breadth in individual local policies. The low score in content is mainly manifested in two aspects. First, Policy Q18, which aims to standardize the planning, construction, handover, and management of CHC service facilities in urban residential areas, is a policy focused on the implementation of specific content and does not cover other aspects, thus resulting in a relatively low score in terms of content. Second, while the policy covers multiple domains, it lacks specificity and depth in critical operational areas. For example, Q15, a policy formulated by the Tianjin Civil Affairs Bureau to promote community-embedded older adult care institutions, focuses on providing integrated services including professional nursing, daily care, psychological support, and in-home services to older adults in need. However, it lacks provisions for digital devices, infrastructure, and internet-based services, which are increasingly integral to modern care ecosystems. This omission of digital components may diminish the effectiveness and user experience of policy implementation.

### 3.4 PMC surface of the central and local CHCPs

**Figure 5:**
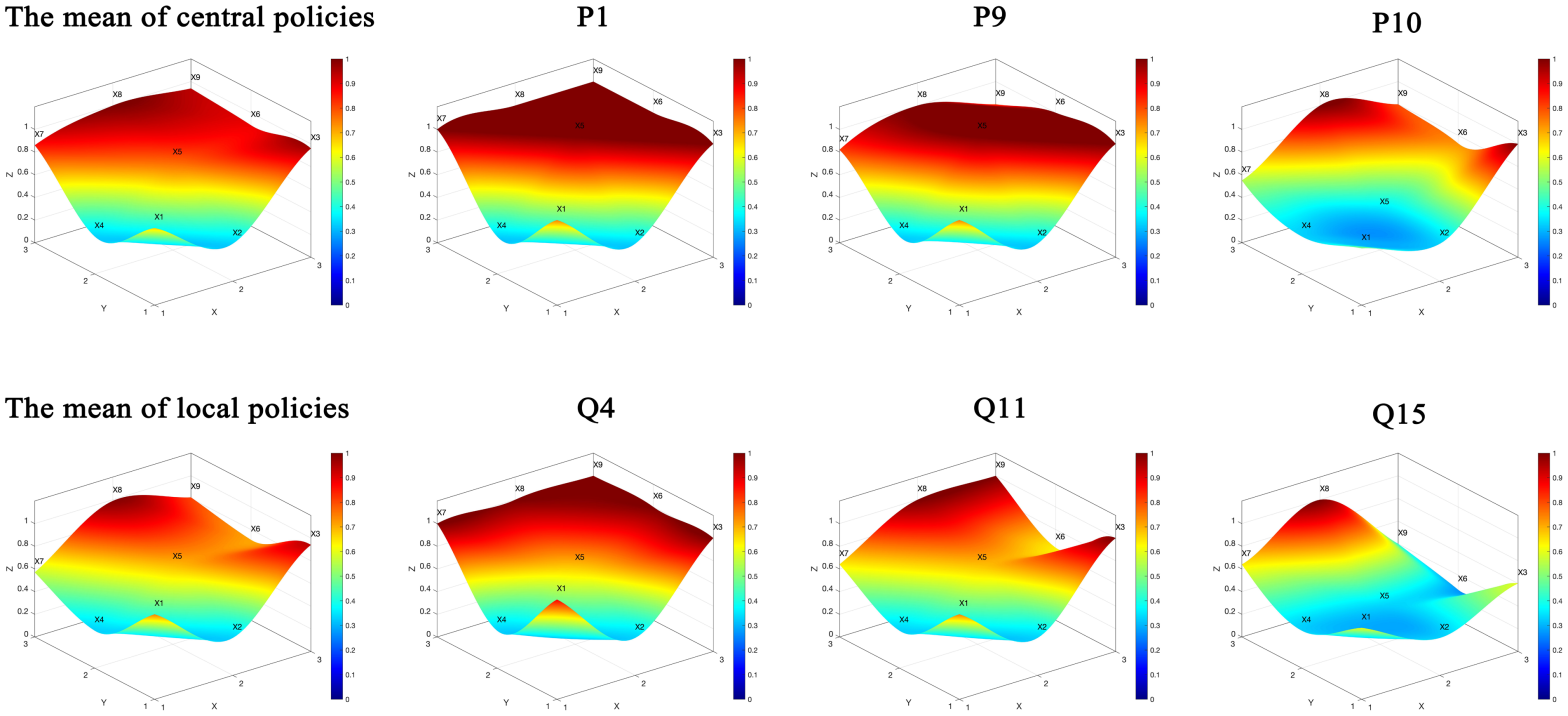
The PMC Index surface of central and local policies (partial).

The PMC surface offers an intuitive visualization of policy consistency across CHCPs. Surface concavity and color intensity correspond to policy quality: convex, darker surfaces indicate higher aggregate scores, while flatter, lighter ones reflect lower performance, as shown in Figure 5.

Based on the calculated PMC Index results, eight representative datasets were selected from both central and local CHCPs to construct comparative PMC surfaces. Central policies include the mean index value, the highest-scoring (P1), lowest-scoring (P10), and mid-range (P9) policies.

Policy P10 is designed to promote the healthy development of older adult care and childcare services and is a specific measure to ensure and improve people’s livelihoods and promote long-term balanced population development. As shown in Figure 5, the concave nature of X1, X2, X4, X5, and X7 indicates that this policy needs further improvement and strengthening in terms of (X1), (X2), (X4), (X5), and (X7). There is a certain gap in the overall coherence of the policy dimensions. Specifically, in terms of (X1), the policy lacks predictive and regulatory functions for community-based home care for older adults, as well as a description and diagnosis of the current situation. Regarding (X7), the comprehensiveness is relatively insufficient, meaning that the policy is highly targeted and specific in its formulation, particularly lacking in areas such as medical care, daily living assistance skills for older adults, and digital intelligence applications.

In contrast, for local CHCPs, four representative datasets—the mean index, along with the highest-scoring (Q4), lowest-scoring (Q15), and mid-range (Q11) policies—were visualized. The lowest-scoring Policy Q15 aims to use the development of community-embedded older adult care service institutions as a new driving force to reform the older adult care service supply method and achieve dual breakthroughs in professional service level improvement and specialization, thereby enhancing humanistic care and meeting the “aging in place” demand. As shown in Figure 5, the concave nature of X1, X2, X4, X5, and X6 indicates that the policy still needs improvement in (X1) and (X4). The significant convexity of X8 shows that the policy focuses on the development of community-embedded older adult care institutions, describing the development tasks, basic characteristics, service functions, and work requirements of these institutions. The policy is highly targeted, with rigorous planning and scientifically formulated plans that meet national strategic needs, resulting in a high score in the policy evaluation dimension (X8).

The comparison of quantitative analysis reveals a nuanced pattern of policy alignment and divergence across governance tiers, as detailed below: (1) Policy Nature (X1) and Policy Limitation (X4) show a shared systemic gap. Both central and local policies exhibit significant weaknesses in these dimensions. The consistently low scores reflect a system-wide deficiency in descriptive, diagnostic, feedback, and long-term structure, undermining policy adaptability and sustainability. (2) Policy Audience (X3), Policy Evaluation (X8), and Policy Area (X9) show a strong vertical alignment. High scores in these variables across both tiers indicate successful top-down transmission of core strategic priorities, ensuring consensus on target beneficiaries, performance assessment frameworks, and sectoral coverage. (3) Policy Type (X2) and Policy Content (X7) show a functional divergence. A clear functional division of labor is observed. Central policies predominantly employ macro-level directives (X2), while local counterparts focus on basic and specific measures. This leads to a content (X7) misalignment, where local policies, though more operational, often lack the comprehensiveness of central guidelines. (4) Policy Instrument (X5) shows a critical local shortcoming. This dimension represents a pronounced local-level deficiency. The underutilization and limited diversity of policy tools significantly constrain the effectiveness and evidence-based nature of local implementation.

In summary, the CHCPs PMC Index is characterized by strategic consistency at the central and local levels but is hampered by operational misalignment and instrumental gaps at the local level, which ultimately restrict policy efficacy. These findings collectively underscore a complementary policy architecture: central policies provide strategic direction, while local implementations operationalize context-specific solutions within a nationally aligned framework.

## 4 Discussion and implications

### 4.1 Discussion of CHCPs in Japan and South Korea

The global confluence of population aging, rising chronic disease prevalence, and escalating healthcare costs has intensified the demand for sustainable older adult care models. In response, countries such as Japan and South Korea have pioneered systemic innovations centered on community-based care, offering valuable references for China’s ongoing policy development (38).

Japan is renowned for its relatively high degree of population aging, presenting a unique scenario in the field of older adult care. Japan introduced the public long-term care insurance (LTCI) system in 2000. First, its long-term institutional design, supported by sustainable funding and stable service delivery mechanisms, ensures continuity in older adult care while establishing predictable pathways through legislative and policy stability. Second, LTCI integrates multidimensional services such as financial insurance, healthcare, psychosocial support, and assistive devices into needs-based personalized packages. This modular approach enables both precision in service delivery and flexibility in implementation. Finally, the system employs a polycentric governance model that engages government, market actors, and civil society in collaborative community-based care. This multi-stakeholder framework enhances resource allocation efficiency and mitigates state fiscal burdens through shared responsibility, thereby institutionalizing a sustainable older adult care ecosystem (39–43).

South Korea has institutionalized the concept of “community care” as a strategic pivot in older adult healthcare, shifting from a hospital-centric treatment paradigm toward a community-embedded care model. This transition is operationalized through tailored community-based interventions, including structured outreach programs that prioritize geographically coordinated implementation (44, 45). Three distinctive features characterize this approach. First, the Korean government has strategically established senior centers as community hubs that provide integrated health-promotive services alongside social and recreational activities. These centers function through multi-level partnerships that enhance resource coordination across regional jurisdictions, demonstrating measurable benefits in physical health outcomes among older adults. Second, the healthcare system integrates chronic disease management into primary care through standardized registration and monitoring programs. Grounded in the chronic care model, this systematic approach strengthens preventive care capacity at the community level (46). Third, policy targeting explicitly prioritizes socioeconomically vulnerable populations, ensuring that community care mechanisms address structural inequities in health access. This equity-oriented design enhances the inclusiveness of service delivery while mitigating care disparities in aging populations (47, 48).

By contrast, the policy for CHC in China has great room for improvement. In contrast to Japan’s legislatively anchored long-term care insurance system, Chinese policies remain oriented toward short- to medium-term planning, lacking the sustained fiscal and institutional mechanisms necessary for long-term care security. Regarding policy audiences, while China has established relatively comprehensive CHC frameworks, eligibility criteria remain ambiguously defined compared to Korea’s explicitly prioritized vulnerable groups, limiting the inclusion of populations such as persons with disabilities. Furthermore, despite relatively well-developed policy content, China’s approach lacks the service integration observed in Japan’s multidimensional care packages or Korea’s chronic disease management systems. Specifically, senior centers in China continue to prioritize physical health services, with insufficient attention to psychosocial support.

### 4.2 Implications

Based on the multidimensional empirical findings presented above and the analysis of international experience, the study attempts to propose several practical implications to enhance the implementation and optimization of China’s community-based home care policies (CHCPs).

First, in the aspect of policy audience, although existing policy already involves multiple stakeholders, an effective policy adjustment for CHC must prioritize a diversified provider base, actual beneficiaries, clearly defined roles, and enhanced inter-agency collaboration. Current challenges, such as some people with disabilities also needing to be classified as beneficiaries of the CHCPs, and unclear service eligibility that leads to inequitable access for older adults with similar needs (49), underscore that policy design must not only refine target beneficiary groups to guarantee universal, needs-based access (49) but also explicitly define the responsibilities of all participating entities to improve implementation. Finally, it is essential to foster collaboration among service providers by supporting their equal and effective participation, thereby establishing a cooperative supply framework based on broad social engagement and collective action.

Second, regarding policy nature, the design of CHCPs should establish various feedback mechanisms and enhance monitoring throughout implementation to better diagnose the target issue. The institutional questions, and at times technical issues that have often been identified in policymaking stages such as policy formulation, are also likely to unfold in relation to policy monitoring practice. Policy monitoring is often seen as a crucial ingredient of policy evaluation (37). By incorporating diverse social interests via open communication and monitoring platforms, policy acceptability can be improved, and quality can be evaluated, while ongoing content optimization helps reduce implementation resistance and increase effectiveness.

Third, regarding policy limitations, CHCPs must incorporate a long-term temporal perspective in their design. For macro-level policies, the extended duration from promulgation to implementation necessitates an extended timeframe to avoid efficacy loss, whereas specific policies require sufficient time to yield measurable outcomes. Given that most elders over 65 are prone to the adverse side effects of various chronic diseases such as hypertension, cardiovascular disease, diabetes, and dementia, an integrated long-term care model addressing both physical and psychological needs is essential for effective older adult policy design (50–52). Consequently, policy timeframes should account for the needs of the target population strategically and be aligned with their respective content and objectives.

Fourth, regarding the content of policy, policy design requires greater breadth and depth to fully encompass the multifaceted nature of community home care. Research indicates that community-based home care encompasses a full spectrum of health and social services from community-based to institutional settings (49). In the process of CHC, a multidisciplinary professional team comprising geriatric specialists, nurses, and other health professionals is crucial for addressing the specific needs of older patients (53). Moreover, the integration of digital technologies has significantly improved older adult care by optimizing resource allocation and enabling personalized care, thereby enhancing the quality of life for older adults among other (54, 55). Based on the Policy Content analysis, this entails targeted enhancements in critical dimensions such as reasonable financial support (49), long-term care talent cultivation, spiritual comfort, and electronic product development (56), in which policy content should be strengthened to establish a more comprehensive and effective service system.

### 4.3 Conclusion

As the first study to integrate CHCPs with a rigorous multidimensional empirical analysis, this research provides a new perspective that addresses a significant literature gap. By doing so, it not only supplies empirical evidence to guide local governments in enhancing their policies but also offers actionable insights for the design and development of future community-based home care enterprises.

First, the analysis reveals a distinct top–down governance structure with strong longitudinal consistency between central and local policies. However, significant horizontal disparities persist across local implementation capacities. Specifically, four of the nine policy dimensions assessed policy nature, target audience, timeliness, and content emerge as critical areas requiring improvement.

Second, the research makes significant advancements by expanding the application of the PMC Index model to aging policy analysis. The study developed a comprehensive evaluation framework capable of conducting simultaneous longitudinal and horizontal consistency assessments, thereby addressing the literature’s predominant focus on single-dimensional analyses. The introduced approach effectively captures previously obscured inter-tier dynamics and implementation gaps, enhancing the methodological robustness of policy consistency evaluation in multi-level governance contexts.

Finally, based on the evident results and the experience of the other countries, the findings offer concrete guidance for policymakers. To address identified deficiencies, the paper proposes: establishing dynamic policy adjustment mechanisms responsive to evolving older adult needs; clarifying responsibility distributions among diverse audience; developing differentiated service packages for diverse older adult cohorts; and integrating digital health technologies with traditional care models These evidence-based recommendations aim to enhance policy coherence and practical effectiveness in China’s aging-care system.

### 4.4 Limitations

This study acknowledges several limitations that warrant consideration. First, the restricted timeframe (2021–2024), while ensuring policy timeliness, necessarily excludes earlier foundational policies that may continue to shape current implementation. This temporal constraint potentially limits our understanding of policy evolution trajectories and long-term effectiveness, particularly for policies with extended implementation horizons. The identified consistency patterns thus represent a dynamic process in the recent 5 years rather than a longer assessment.

Second, the sample’s exclusive focus on provinces with the most advanced aging populations limits the comprehensiveness of the findings. While the 10-province framework provides valuable insights into policy responses in critically aged regions, it lacks critical variation from early and moderately aging regions, thus limiting how representative the findings are for understanding policy dynamics across the full spectrum of China’s aging trajectory.

Third, the exclusive focus on China’s policy framework, though providing depth to a critical case, affects the comprehensiveness and generalization of findings. As aging is a global issue with numerous policy responses worldwide, analyzing and comparing CHCPs solely within China provides a confined perspective, restricting the breadth of the analytical viewpoint.

Future research will address these gaps by, first, conducting longitudinal follow-up studies to assess the long-term effects and implementation dynamics of these policies. Second, to expand the sample size to the different level of aging regions to provide more comprehensive analysis in diverse policies. Third, it will expand the applicability of the PMC Index model by incorporating policy sample from other countries, thereby facilitating a cross-national comparative analysis and adding a broader dimension to CHCPs assessment.

## Data Availability

The original contributions presented in the study are included in the article/supplementary material, further inquiries can be directed to the corresponding author.
